# The Impact of Different Hepatitis B Virus Serological Statuses on the Safety of Different Chemotherapy Regimens in Female Breast Cancer Patients: A Within-Subject Longitudinal Study

**DOI:** 10.3390/cancers17213574

**Published:** 2025-11-05

**Authors:** Zhao-Xing Li, Dong-Li Liu, Lei Hu, Bai-Qing Peng, Xiu-Quan Qu, Li-Yuan Mu, Xiao-Chun Cheng, Pu Qiu, Yu-Xuan Huang, Xi-Rui Li, Ling-Quan Kong

**Affiliations:** 1 Department of Breast and Thyroid Surgery, The First Affiliated Hospital of Chongqing Medical University, Chongqing 400016, China; 2022150054@stu.cqmu.edu.cn (Z.-X.L.);; 2 Information Center, The First Affiliated Hospital of Chongqing Medical University, Chongqing 400016, China

**Keywords:** hepatitis B virus, breast cancer, chemotherapy safety, hepatotoxicity, HBV reactivation

## Abstract

**Simple Summary:**

Breast cancer patients who are currently or were previously infected with the hepatitis B virus (HBV) face increased risks of liver injury when undergoing chemotherapy. This study examined how different HBV infection statuses affect liver safety during chemotherapy in a large group of over 4500 patients. We found that patients with active HBV infection had the highest risk of liver injury and treatment interruptions, with the risk peaking during specific chemotherapy cycles. Even patients with past infection showed a higher need for treatment changes. These findings highlight the importance of tailored monitoring and preventive antiviral therapy for HBV-affected breast cancer patients to complete chemotherapy safely and effectively.

**Abstract:**

**Background:** Breast cancer patients with hepatitis B virus (HBV) infection or past HBV infection face heightened risks of chemotherapy-induced hepatotoxicity and HBV reactivation (HBVr). This study aims to evaluate the impact of different HBV serological statuses on the safety of chemotherapy regimens based on paclitaxel throughout the treatment cycle. **Methods:** This retrospective cohort study analyzed 4562 female breast cancer patients, categorized into three groups: 366 with HBV infection (HBsAg+), 2529 with past HBV infection (HBsAg−/HBcAb+), and 1667 without HBV infection (control group). The Primary events included liver injury, HBVr, treatment interruption, and laboratory indicator evaluation. Demographic characteristics and periodic laboratory parameters were recorded for within-subject longitudinal analysis. **Results:** Before chemotherapy, the incidence of liver injury was highest in the HBV-infected group (18.2%), intermediate in the past-infection group (13.2%), and lowest in the control group (12.0%). Throughout chemotherapy, the cumulative incidence of liver injury remained highest in the HBV-infected group (83.2%), compared to the past-infection (71.2%) and control (70.9%) groups. Chemotherapy interruption rates followed a similar gradient: 12.4% in the HBV-infected group, 6.9% in the past-infection group, and 5.5% in the control group. HBV-infected patients had a significantly higher risk of hepatotoxicity than controls during cycle 4 (relative risk 1.56, 95% CI 1.06 to 2.29) and cycle 5 (1.28, 1.09 to 1.75). HBVr occurred in 13 patients with HBV-infected. **Conclusions:** HBV serological status significantly impacts chemotherapy safety and treatment interruption. Prophylactic antiviral therapy and intensified monitoring during high-risk cycles (cycle 4 and cycle 5) are critical. These findings underscore the necessity of stratified management for HBV-affected breast cancer patients during chemotherapy.

## 1. Introduction

Breast cancer is the most prevalent malignancy among women, accounting for approximately 32% of new cancer cases and 14% of cancer-related deaths in females according to 2025 US cancer statistics [1]. Meanwhile, the WHO 2024 Global Hepatitis Report [2] indicates that hepatitis B virus (HBV) remains endemic worldwide, affecting approximately 254 million people globally. China, classified as a moderate-to-high endemic region, has a hepatitis B surface antigen (HBsAg) seroprevalence of 5.86% in the general population [3,4]. Notably, breast cancer patients in China have even higher rates of current and past HBV infection. Some studies report HBV infection rates of 7.9% and 55.2% past HBV infection among women with breast cancer [5], which imposes a dual disease burden.

Chemotherapy remains a cornerstone of breast cancer treatment, significantly improving disease control, survival, and quality of life, but its associated adverse effects merit clinical attention. HBV-infected patients often have compromised baseline liver function, and commonly used chemotherapeutic agents (e.g., taxanes, anthracyclines) exert additional hepatotoxicity, frequently necessitating treatment delays or discontinuations [6]. Moreover, chemotherapy may trigger HBV reactivation (HBVr) in chronic carriers, which can progress to fulminant hepatitis or acute liver failure with high mortality rates, severely undermining outcomes [7,8]. A meta-analysis of 26 studies [9] reported median HBVr rates of 25% (4–68%) vs. 4.1% (0.9–31.4%) in HBsAg+/HBcAb+ solid tumor patients without vs. with antiviral prophylaxis, respectively. Among breast cancer patients, reactivation rates are particularly high (20–41%) without preventive measures [10].

Although existing studies have explored HBV’s impact on chemotherapy toxicity [11], data on differential effects across regimens and treatment cycles—especially in patients with past HBV infection (HBsAg−/HBcAb+)—remain limited. This study aims to analyze the impact of different HBV serological statuses on the safety of chemotherapy regimens based on paclitaxel in breast cancer patients throughout the treatment cycle.

## 2. Materials and Methods

### 2.1. Patients

This study enrolled 6515 female breast cancer patients diagnosed at the Chongqing Breast Cancer Center (Chongqing is a municipality in China with a population of 30 million) who received chemotherapy between September 2012 and September 2020. All pathological diagnoses were confirmed by expert pathologists at the Chongqing Pathology Center. A total of 4562 breast cancer women were included in this study after exclusion.

**Figure 1:**
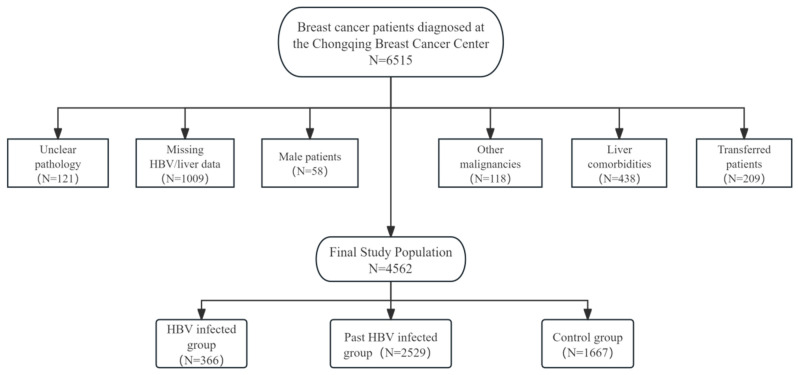
Study design flow chart and exclusion and grouping.

Exclusion criteria included: (1) unclear pathological diagnosis; (2) incomplete baseline HBV serological markers (HBsAg) or liver function data before initial chemotherapy; (3) male patients; (4) concurrent other malignancies; (5) comorbidities potentially affecting liver function (hepatitis C, decompensated liver disease, or cirrhosis); (6) transfer to other hospitals during chemotherapy. As detailed in Figure 1, the final cohort included 366 HBV-infected breast cancer patients, 2529 past HBV-infected breast cancer patients, and 1667 breast cancer patients with no history of HBV infection as observation and control groups, respectively. For the final analytical cohort, the data completeness for longitudinal laboratory parameters (e.g., ALT, AST) was exceedingly high, with a missing rate of less than 1% across all chemotherapy cycles. Demographic characteristics and periodic laboratory parameters were collected for within-subject longitudinal analysis (Figure 1).

This study was approved by the Ethics Review Committee of the First Affiliated Hospital of Chongqing Medical University (approval number: K2024-227-01) and conducted in accordance with the Declaration of Helsinki. As a retrospective investigation using observational data without interference in public behavior, the requirement for written informed consent was waived by the Ethics Committee in compliance with local/national regulations.

### 2.2. The Chemotherapy Regimens

The primary chemotherapy regimens in this study were paclitaxel-based, including:

TEC: docetaxel, anthracyclines, and cyclophosphamide every 3 weeks for 6 cycles.
EC-T(H): epirubicin and cyclophosphamide for 4 cycles, followed by docetaxel with or without trastuzumab for 4 cycles (all cycles administered every 3 weeks).
TC: docetaxel and cyclophosphamide every 3 weeks for 4 or 6 cycles.

A small number of patients received other regimens. All treatments were administered in accordance with the National Comprehensive Cancer Network (NCCN) guidelines [12].

### 2.3. Detection Methods and Criteria

HBV serological markers were systematically recorded for all patients before chemotherapy, with high-sensitivity HBV DNA testing performed for suspected reactivation cases. HBV serological testing used time-resolved fluoroimmunoassay (TRFIA) for HBsAg/HBsAb and ELISA for HBeAg/HBeAb/HBcAb, following the standardized protocols with rigorous quality control. Diagnostic classifications were: (1) HBV-infected (HBsAg+); (2) past HBV infection (HBsAg−/HBcAb+); (3) control group (remaining serological profiles). Serum HBV DNA quantification utilized the Roche COBAS TaqMan assay (sensitivity: 20 IU/mL; range: 20–69,000,000 IU/mL) (Roche Shanghai, China). HBVr was defined as a ≥10-fold increase in HBV DNA from baseline [13,14]. Liver function assessment adhered to the NCI CTCAE v5.0 criteria, with hepatotoxicity of any grade defined as liver injury. Specifically, the occurrence of ALT > 35 U/L (exceeding the upper limit of normal), AST > 35 U/L, or TBil > 21 μmol/L at any point during treatment was considered Grade 1 or higher hepatotoxicity [12].

### 2.4. Statistical Methods

Microsoft Excel 2019 and IBM SPSS Statistics v26 were used for the data record and analysis. The continuous variable was described by medians (interquartile) or means ± standard deviations. The Shapiro–Wilk method or Kolmogorov–Smirnov method were used for normal detection. Non-normally distributed continuous variables were analyzed using nonparametric tests. The categorical variable was described by numbers (proportions) and tested by Chi-square test or Fisher’s exact test. To address potential type I errors from multiple comparisons, sensitivity analyses with Bonferroni correction were performed. Statistical significance was defined as *p*-value < 0.05. Generalized estimating equations (GEE) were employed to analyze the incidence of liver injury. The longitudinal changes in continuous liver function parameters (ALT, AST, and TBil) were analyzed using linear mixed-effects models (LMMs), a “composite symmetry” covariance structure was selected for the repeated measurement of chemotherapy cycles, this model has advantages in simplicity, stability and interpretability. Fixed effects include age, HBV serological status, chemotherapy regimen, chemotherapy cycle and their interaction terms. The selection of these covariables is based on their clinical relevance and previous literature evidence, aiming to evaluate their core impact on liver function trajectories [5,6,7,8]. Due to the right-skewed distribution commonly observed in biochemical markers, all values were subjected to a natural logarithmic (ln) transformation to meet the model’s assumptions of normality and homoscedasticity. The assumptions of the linear mixed-effects models (normality and homoscedasticity of the residuals) were verified by inspection of residual plots after model fitting, which confirmed that the ln-transformation successfully addressed these assumptions. The results of the models for these parameters are presented on the log-transformed scale. For ease of clinical interpretation, these estimates were subsequently back-transformed (exponentiated) and reported as percentage changes relative to the baseline reference. We have centered the continuous variables and examined the variance inflation factor (VIF), confirming that there is no severe multicollinearity (VIF < 5). Due to the low missing rate (<1%) in this study and the fact that the linear mixed-effects model can provide effective estimates under the assumption of random missing when using maximum likelihood estimation, we employed the complete analysis method.

## 3. Results

Baseline characteristics of the cohort are presented in Table 1, including 366 HBV-infected 2529 past HBV-infected and 1667 control group female breast cancer patients. Pre-chemotherapy comparisons showed no significant differences among groups in BMI, activated partial thromboplastin time, thrombin time, total leukocyte count, or tumor size (T-staging). The past HBV infection group was older than the control group. (50, [44 to 58] vs. 49, [42 to 55] years), while the HBV-infected group was the youngest (48, [43 to 55] years) (*p* < 0.05).

The HBV-infected group had significantly elevated alanine aminotransferase (ALT), aspartate aminotransferase (AST), total bilirubin (TBil), prothrombin time (PT), and liver injury incidence (65 cases, 18.2%) compared to other groups (*p* < 0.05). The past HBV-infected group had higher ALT, AST, and ALP levels than controls (*p* < 0.05). However, the rate of liver injury incidence (314 cases, 13.2%) is not statistically different from controls (191 cases, 12.0%) (*p* > 0.05).

Table 2 presents the cumulative incidence rate of clinical events during chemotherapy stratified by HBV serological status. The HBV-infected group had the highest rate of moderate-to-severe myelosuppression in total chemotherapy cycles (10.9%), though this was not statistically significant (*p* > 0.05). This group also had significantly higher rates of chemotherapy-associated liver injury (83.2%), treatment interruption (12.4%), and HBV reactivation (13 cases, 3.6%) than other groups (*p* < 0.05). The past HBV-infected group had rates of myelosuppression and liver injury comparable to those in the control group (*p* > 0.05), but showed a higher rate of treatment interruption (6.9% vs. 5.5%, *p* < 0.05). No cases of HBVr were observed in this group.

Table 3 summarizes the laboratory parameters—including ALT, AST, TBIL, liver injury incidence, PT, thrombin time (TT), activated partial thromboplastin time (APTT), and total leukocyte count—at baseline and during peak-risk chemotherapy cycles, stratified by HBV serological status and regimen.

For the TEC regimen, the HBV-infected group reached peak median ALT (40 U/L) and AST (29 U/L) at cycle 4, with 60% (138/230) of patients developing liver injury, all significantly elevated from baseline (*p* < 0.05). Concurrently, TBIL, TT, and pre-cycle leukocyte counts declined to their nadir at cycle 5 (e.g., leukocytes: 4.16 × 10^9^/L in the HBV-infected group), which were also significantly lower than baseline (*p* < 0.05). The past-infection group showed similar trends but with lower magnitudes of change (Table 3).

A similar pattern was observed with the EC-T(H) regimen, where liver injury incidence peaked at cycle 6. At this point, 71.4% (59/83) of HBV-infected patients developed liver injury, accompanied by significantly elevated median ALT (41 U/L) and AST (33 U/L) (*p* < 0.05 vs. baseline). These values were notably higher than those in the past-infection group (51.0% liver injury, ALT: 34 U/L, AST: 27 U/L; *p* < 0.05 for intergroup differences). Nadirs for TBIL and leukocyte counts were observed at cycle 5.

For the TC regimen, the most pronounced intergroup differences occurred at cycle 4, with the HBV-infected group exhibiting the highest median ALT (56 U/L) and AST (39 U/L) among all regimens (*p* < 0.05 vs. baseline and past-infection group).

Across all three regimens, PT demonstrated significant cycle-specific variations from baseline (*p* < 0.05), whereas APTT showed no consistent significant changes. Complete longitudinal data are available in Supplementary Table S1.

The linear mixed-effects models and generalized estimating equations, which were fitted to ln-transformed ALT, AST, and TBil values, identified the chemotherapy cycle as the predominant factor driving longitudinal changes in liver function, leukocyte count, and liver injury risk (all *p* < 0.001). A significant interaction between cycle and HBV status (*p* < 0.001) indicated that HBV-infected patients experienced distinctly different trajectories of liver injury. HBV infection and age were also independent significant predictors of worse hepatic and coagulation outcomes. In contrast, the specific chemotherapy regimen had a comparatively minor influence on these parameters. The significant random intercept confirmed substantial baseline variability among individuals, underscoring the necessity of a longitudinal study design (Table 4).

Using linear mixed-effects models with patients in the control group at cycle 1 as the reference, we analyzed longitudinal changes in liver function parameters after natural logarithm transformation. The model estimates were subsequently exponentiated to express the effects as percentage changes relative to this baseline reference. This analysis revealed that HBV-infected patients exhibited a significant initial decline in ALT levels by −9.7% (−15.6%, −3.3%) at cycle 2, followed by a pronounced peak increase of +16.7% (+8.9%, +25.0%) at cycle 4. A parallel surge was observed in AST, which reached its zenith at the same cycle with an increase of +14.4% (+8.6% to +20.3%). In contrast, past-infected patients showed more stable ALT profiles, with their most notable change being a reduction at cycle 3 (−4.7%; −8.2% to −1.0%).

The risk of liver injury, derived from the same model framework using generalized estimating equations with the identical reference group, showed a markedly elevated risk for HBV-infected patients at cycle 4 (1.56, 1.06 to 2.29; *p* = 0.025) and cycle 5 (1.28, 1.09 to 1.75; *p* = 0.031). The differences in trajectories between HBV-infected (Table 5) and past-infected (Table 6) groups, both compared to the non-infected, pre-chemotherapy baseline, underscore the distinct impact of active HBV infection on chemotherapy-induced hepatotoxicity.

## 4. Discussion

Breast cancer represents a substantial health burden in Europe and North America. In recent years, rates have also risen steadily in the Western Pacific region, making breast cancer the most common cancer among women in China [15,16]. Previous studies report that the prevalence of current and past HBV infection among newly diagnosed breast cancer patients in China is 7.9% and 55.1%, respectively [5]. However, the dynamic impact of HBV status on chemotherapy safety throughout the treatment cycle remains poorly characterized. Our within-subject longitudinal analysis, employing appropriate statistical modeling to account for repeated measures, clearly demonstrates that active hepatitis B virus (HBV) infection imposes a significant additional burden on chemotherapy safety in breast cancer patients. The key findings are that (1) HBV serological status is an independent predictor of hepatotoxicity trajectories, (2) the risk of liver injury evolves dynamically throughout chemotherapy, with specific peak risk periods, and (3) this leads to a clinically significant increase in treatment interruptions. These findings underscore the necessity for a stratified and dynamic management approach.

Breast cancer treatment primarily involves surgery, supplemented by adjuvant therapies tailored to tumor biology and patient characteristics. Chemotherapy is one of the most widely used and effective adjuvant treatment methods [17]. Commonly administered chemotherapeutic agents—such as docetaxel, anthracyclines, cyclophosphamide, and fluorouracil—exhibit varying degrees of hepatotoxicity, frequently contributing to liver injury. In breast cancer patients with chronic HBV infection, chemotherapy may trigger reactivation of latent or quiescent HBV, leading to severe liver injury and adversely affecting prognosis. Chemotherapeutic drugs, along with certain biologics, can promote HBVr through multiple mechanisms: Anthracyclines and fluorouracil suppress lymphocyte function, inhibiting tumor necrosis factor-β (TNF-β) and interferon-γ (IFN-γ) pathways, thereby reducing antiviral cytokine production and facilitating HBVr [18]. Cyclophosphamide impairs lympho B cell activation, proliferation, and differentiation, further increasing HBVr risk [19].

In line with major guidelines and expert consensus, HBsAg-positive patients should initiate prophylactic antiviral therapy 2–4 weeks before chemotherapy and continue treatment to prevent HBVr [4,20,21]. Among 366 HBsAg-positive breast cancer patients receiving chemotherapy at our institution, 13 cases (3.6%) exhibited HBVr. This incidence aligns with prior studies [9,10], likely attributable to routine hepatoprotective measures and prophylactic antiviral use.

Following HBV entry into the host, the viral nucleic acid can integrate into the nuclear genome of hepatocytes, forming covalently closed circular DNA (cccDNA) [22], which persists long-term in liver cells and accounts for both the difficulty in achieving complete viral eradication and the large population with past HBV infection. As only a very small number of past HBV infection patients who might have developed HBVr underwent selective HBV DNA testing, the sample size was limited, and no obvious HBVr was observed. However, some studies suggest that for patients with past HBV infection who have moderate-to-high risk factors for HBVr [11,23], enhanced monitoring should be implemented, and prophylactic antiviral therapy should be considered when clinically indicated. A noteworthy finding is that the past-infection group did not exhibit a significantly higher cumulative incidence of liver injury than the control group, despite being at theoretical risk. This is likely a testament to the effectiveness of conventional hepatoprotective strategies routinely employed in patients with underlying liver conditions. However, the absence of a statistical difference in ‘laboratory-defined’ hepatotoxicity stands in contrast to their elevated rate of chemotherapy interruptions (6.9% vs. 5.5%). This discrepancy could be attributed to the generally poorer baseline health status, a higher burden of comorbidities, or suboptimal health behaviors among patients with past HBV infection. Therefore, even in the absence of overt laboratory toxicity, patients with past HBV infection warrant continued attention and monitoring, especially for patients with high-risk factors [11].

Chemotherapy must be administered as scheduled to achieve optimal therapeutic outcomes, as premature discontinuation may increase the risk of recurrence [24,25]. Multiple studies have demonstrated that patients who complete their chemotherapy regimens exhibit significantly higher long-term survival rates compared to those with treatment interruption or delays [26,27]. Drug-induced toxicity, particularly hepatotoxicity, is a major driver of treatment interruption. Our study revealed a graded increase in chemotherapy interruption rates that paralleled the severity of HBV-related liver vulnerability: highest in the HBV-infected group (12.4%), followed by the past-infection group (6.9%), and lowest in the control group (5.5%). This gradient suggest that liver injury, which was prevalent and severe in the HBV-infected group as demonstrated by our longitudinal models, may be a primary driver of treatment disruption in these patients. While factors like poor compliance or financial constraints cannot be entirely ruled out [28,29], the inclusion of a control group helps to baseline the rate of interruptions from other common toxicities (e.g., severe gastrointestinal reactions, neurotoxicity, or cardiotoxicity [30,31,32]). The excess interruption observed in the HBV-affected groups is therefore most plausibly attributed to HBV-associated hepatotoxicity.

Furthermore, the immunosuppressive effects of chemotherapy can promote HBV replication [33], potentially creating a vicious cycle of escalating liver injury and further increasing the risk of interruption due to hepatitis flare or, in severe cases, fulminant liver failure [34].

Current guidelines and expert consensus recommend initiating prophylactic antiviral therapy 2–4 weeks before immunosuppressive or cytotoxic chemotherapy in HBsAg-positive patients [4,20,21]. Our study adds a critical temporal dimension to this recommendation. In chemotherapy regimens based on paclitaxel, the occurrence of liver injury in patients with HBV infection is significantly higher than that in the control group in cycle 4 (1.56, 1.06 to 2.29; *p* = 0.025) and cycle 5 (1.28, 1.09 to 1.75; *p* = 0.031). It is worth noting that although no statistical difference in liver injury between the population with past HBV-infected and the control group was found in each chemotherapy cycle, univariate analysis still suggested that there was a peak in the occurrence of liver injury in this group of people in the 4th cycle of the TEC regimen and the 6th cycle of the EC-T regimen. The finding that risk peaks provides a clear evidence base for intensified, cycle-specific monitoring. We propose that, in addition to universal prophylaxis, HBV-affected patients should undergo intensified liver function and HBV DNA monitoring around these high-risk cycles to enable pre-emptive intervention and potentially prevent treatment-disrupting complications.

This study possesses several notable strengths. First, its within-subject longitudinal design and the application of advanced statistical models (GEE and LMM) allow for a robust analysis of dynamic changes in liver function over time, capturing the temporal pattern of hepatotoxicity risk more accurately than cross-sectional studies. Second, the large sample size (n = 4562) provides substantial statistical power to detect differences across HBV serological subgroups and chemotherapy regimens. Lastly, the identification of regimen-specific and cycle-specific peak risk periods offers concrete, actionable insights for tailoring monitoring and intervention strategies in clinical practice. Although this study provides important data on the safety of chemotherapy in breast cancer patients with current or past HBV infection, several limitations should be acknowledged. The data were derived from a single-center electronic medical record system and were retrospective in nature, which may introduce selection bias and information bias [35,36]. Furthermore, our use of Generalized Estimating Equations (GEEs) to estimate cycle-specific risks does not explicitly account for competing risks, such as treatment discontinuation due to non-hepatic adverse events. Future studies could apply more advanced algorithms, such as bi-phase biclustering, to identify patient subgroups with distinct hepatotoxicity trajectories and enable more personalized monitoring [37]. Additionally, the choice of chemotherapy regimens may have been influenced by clinician preference rather than randomization. While patients received prophylactic antiviral therapy, the specific antiviral agents and treatment durations were not uniformly analyzed in this study. Furthermore, HBV DNA testing was only performed in cases of suspected HBVr, potentially leading to an underestimation of the true HBVr rate.

If feasible, future prospective multicenter cohort studies are warranted to validate these findings. Further research should explore novel biomarkers—such as cccDNA quantification, HBV RNA, or inflammatory cytokines (e.g., IL-6, TNF-α) to predict high-risk populations for HBVr and enable personalized intervention strategies [38,39]. Additionally, the optimal integration of chemotherapy and hepatoprotective strategies in patients with current or past HBV infection remains an area for further investigation.

## 5. Conclusions

This study confirmed that the serological status of hepatitis B is a key factor in the safety of chemotherapy for breast cancer. Patients with HBV infection face the highest risk of liver injury and treatment interruption, and the fourth and fifth cycles of chemotherapy are high-risk cycles, emphasizing the role of strengthening HBV DNA monitoring and antiviral treatment during the peak period of danger. Crucially, even patients with a history of HBV infection showed a heightened risk of chemotherapy disruption, warranting closer attention than previously afforded. The toxicity was not static but crested at specific high-risk cycles (TEC-cycle 4; EC-T[H]-cycle 6). Integrating these tailored approaches is essential to optimize outcomes for HBV-affected breast cancer patients undergoing chemotherapy.

## Figures and Tables

**Table 1:** The baseline characteristics of breast cancer patients cohort with different types of HBV serological markers.

	Control Group (n = 1667)		Past HBV-Infected Group (n = 2529)		HBV-Infected Group (n = 366)		*p* Value
Age (years)	49	(42, 55)	50 *	(44, 58)	48 #	(43, 55)	<0.001
BMI (kg/m^2^)	22.5	(20.3, 24.6)	22.2	(20.2, 24.8)	22.5	(20.4, 24.8)	0.373
ALT (U/L)	15	(11, 23)	16 *	(11, 24)	18 *#	(13, 27)	<0.001
AST (U/L)	17	(14, 21)	18 *	(15, 22)	20 *#	(16, 24)	<0.001
TBIL (μmol/L)	9.4	(7.0, 12.8)	9.7	(7.2, 12.8)	10.2 *#	(7.7, 13.5)	0.010
ALP (U/L)	64	(52, 79)	66 *	(53, 81)	64	(51, 78)	0.007
Abnormal liver function (ALT > 1ULN)	191		314		65 *#		0.007
	12.0%		13.2%		18.2%		
PT (s)	12.8	(12.4, 13.3)	12.8	(12.4, 13.3)	13 *#	(12.6, 13.5)	<0.001
TT (s)	16.5	(16.0, 17.1)	16.6	(16.0, 17.1)	16.6	(16.1, 17.2)	0.025
APTT (s)	35.8	(33.5, 38.0)	35.5	(33.2, 37.8)	35.7	(33.4, 37.5)	0.095
WBC (10^9^/L)	5.54	(4.67, 6.52)	5.54	(4.68, 6.54)	5.46	(4.52, 6.40)	0.151
T Staging							0.881
I	660 (41.3%)		983 (41.1%)		146 (40.7%)		
II	848 (53.0%)		1311 (54.8%)		195 (54.3%)		
III	75 (4.7%)		81 (3.4%)		12 (3.3%)		
IV	17 (1.1%)		19 (0.8%)		6 (1.6%)		

* *p* < 0.05 (VS control group), # *p* < 0.05 (VS Past HBV-infected group). Note: Data are presented as median (interquartile range) for continuous variables and n (%) for categorical variables, unless otherwise specified.

**Table 2:** Differences in clinical event occurrences among HBV serological groups during chemotherapy.

	Control Group		Past HBV-Infected Group		HBV-Infected Group	*p* Value
Moderate-to-severe myelosuppression	135		215		40	0.211
	8.1%		8.5%		10.9%	
Cumulative incidence rate of liver injury	1181		1795		305 *#	0.007
	70.9%		71.2%		83.2%	
Chemotherapy interruption	92		173 *		45 *#	<0.001
	5.5%		6.9%		12.4%	
Hepatitis B virus reactivation					13	
					3.6%	

* *p* < 0.05 (VS control group), # *p* < 0.05 (VS Past HBV-infected group).

**Table 3:** Laboratory parameters and liver injury incidence at baseline and peak-risk chemotherapy cycles, stratified by HBV serological status and regimen.

Chemotherapy Regimen	Cycles of Chemotherapy	Serological Status of HBV	ALT (U/L)	AST (U/L)	TBiL (μmol/L)	Abnormal Liver Function	PT(s)	TT(s)	APTT(s)	WBC (10^9^/L)
TEC (2731, 59.8%)	1	HBV-infected group	19 (13, 28)	20 (16, 25)	10.5 (8.0, 13.4)	44	13.0 (12.6, 13.6)	16.6 (16.1, 17.1)	35.7 (33.1, 37.8)	5.51 (4.48, 6.47)
						18.7%				
		Past HBV-infected group	17 (12, 25)	18 (15, 22)	9.9 (7.3, 12.9)	221	12.8 (12.4, 13.3)	16.6 (16.0, 17.2)	35.5 (33.2, 37.8)	5.57 (4.67, 6.55)
						14.1%				
		Control group	16 (11, 24)	18 (15, 22)	9.7 (7.0, 13.0)	133	12.8 (12.4, 13.3)	16.5 (16.0, 17.1)	35.8 (33.5, 38.1)	5.56 (4.66, 6.60)
						13.0%				
	4	HBV-infected group	40 * (27, 55)	29 * (22, 41)	7.4 * (5.4, 10.1)	138 *	13.0 (12.5, 13.7)	16.1 * (15.5, 16.7)	36.1 (34.4, 38.9)	4.92 * (3.79, 6.22)
						60.0%				
		Past HBV-infected group	29 * (22, 41)	23 * (19, 31)	7.2 * (5.3, 9.3)	558 *	13.0 (12.5, 13.4)	16.1 * (15.6, 16.5)	35.3 (32.8, 37.5)	5.07 * (4.09, 6.43)
						37.4%				
		Control group	30 * (23, 43)	24 * (19, 31)	7.4 * (5.4, 9.7)	391 *	12.9 (12.4, 13.4)	16.1 * (15.5, 16.5)	36.2 (33.9, 38.0)	5.00 * (4.03, 6.32)
						39.7%				
	5	HBV-infected group	33 * (25, 48)	26 * (21, 33)	6.9 * (4.9, 9.5)	103 *	12.9 (12.5, 13.5)	15.8 * (15.4, 16.5)	35.2 (32.5, 37.5)	4.16 * (3.12, 5.42)
						45.5%				
		Past HBV-infected group	28 * (20, 39)	23 * (18, 30)	6.7 * (4.9, 9.0)	514 *	12.7 * (12.2, 13.1)	16.0 * (15.5, 16.5)	35.0 * (33.0, 37.0)	4.42 * (3.36, 5.77)
						35.8%				
		Control group	29 * (21, 41)	23 * (18, 31)	6.7 * (4.9, 9.0)	375 *	12.7 * (12.2, 13.1)	15.9 * (15.4, 16.5)	35.6 (33.2, 37.6)	4.37 * (3.34, 5.85)
						38.9%				
EC-T(H) (1139, 25.0%)	1	HBV-infected group	18 (14, 25)	20 (16, 22)	9.7 (7.6, 13.2)	15	13.0 (12.5, 13.4)	16.6 (16.1, 17.2)	35.8 (33.8, 37.2)	5.44 (4.62, 6.20)
						16.0%				
		Past HBV-infected group	15 (11, 23)	17 (14, 21)	9.2 (6.9, 12.3)	67	12.9 (12.4, 13.3)	16.5 (16.0, 17.1)	35.6 (33.3, 37.8)	5.52 (4.65, 6.49)
						10.5%				
		Control group	15 (11, 23)	17 (14, 21)	9.0 (6.8, 11.7)	54	12.8 (12.4, 13.2)	16.5 (15.9, 17.1)	35.7 (33.3, 37.7)	5.38 (4.54, 6.30)
						12.4%				
	4	HBV-infected group	35 * (27, 47)	28 * (22, 38)	7.2 * (4.6, 9.0)	46 *	13.3 (13.0, 13.9)	16.0 * (15.7, 16.6)	34.2 (33.3, 36.0)	4.91 * (4.05, 6.22)
						53.1%				
		Past HBV-infected group	30 * (22, 42)	25 * (20, 32)	6.5 * (4.6, 8.6)	238 *	13.0 (12.6, 13.5)	16.1 * (15.7, 16.7)	35.1 (32.8, 37.5)	4.67 * (3.77, 5.75)
						40.5%				
		Control group	28 * (20, 44)	24 * (19, 33)	6.2 * (4.5, 8.7)	145 *	13.1 (12.6, 13.6)	15.9 * (15.4, 16.4)	35.2 (33.3, 37.9)	4.47 * (3.76, 5.61)
						36.2%				
	5	HBV-infected group	37 * (27, 52)	30 * (24, 40)	6.4 * (4.9, 8.5)	53 *	13.2 (12.5, 13.6)	16.4 (16.0, 16.8)	33.9 (33.3, 36.5)	4.47 * (3.52, 5.77)
						62.2%				
		Past HBV-infected group	32 * (23, 47)	26 * (21, 36)	6.6 * (4.7, 8.6)	269 *	12.9 (12.5, 13.3)	16.2 * (15.7, 16.6)	35.0 (32.8, 36.9)	4.36 * (3.44, 5.52)
						46.3%				
		Control group	30 * (21, 46)	25 * (20, 33)	6.4 * (4.6, 8.6)	161 *	12.9 (12.5, 13.3)	16.0 * (15.4, 16.6)	35.3 (32.9, 37.9)	3.93 * (3.20, 5.04)
						40.2%				
	6	HBV-infected group	41 * (30, 63)	33 * (26, 42)	7.5 * (5.7, 9.9)	59 *	13.3 (13.0, 13.5)	16.0 (15.6, 16.6)	34.5 (33.1, 36.0)	4.57 * (3.68, 5.74)
						71.4%				
		Past HBV-infected group	34 * (26, 48)	27 * (21, 35)	7.6 * (5.7, 10.0)	297 *	13.0 (12.5, 13.4)	16.2 * (15.7, 16.7)	35.1 (33.2, 37.3)	4.94 * (4.12, 6.10)
						51.0%				
		Control group	34 * (25, 49)	27 * (22, 34)	7.8 * (5.9, 10.1)	208 *	12.8 (12.5, 13.5)	16.0 * (15.6, 16.7)	35.6 (33.8, 38.4)	4.78 * (4.02, 6.03)
						50.4%				
TC (217, 4.8%)	1	HBV-infected group	24 (13, 31)	20 (15, 23)	9.6 (7.2, 15.6)	1	13.0 (12.5, 13.4)	16.6 (16.1, 17.2)	35.8 (33.8, 37.2)	6.33 (5.08, 6.90)
						11.1%				
		Past HBV-infected group	16 (12, 23)	19 (16, 23)	9.3 (6.9, 13.1)	26	12.8 (12.3, 13.1)	16.7 (16.0, 17.2)	35.1 (33.7, 37.6)	5.58 (4.66, 6.42)
						18.6%				
		Control group	16 (12, 21)	17 (15, 22)	8.8 (6.7, 11.3)	11	12.9 (12.4, 13.4)	16.4 (16.0, 17.1)	35.8 (33.7, 38.1)	5.57 (4.74, 6.74)
						11.7%				
	4	HBV-infected group	56 * (44, 57)	39 * (28, 53)	11.1 (9.4, 14.2)	6 *	13.0 (12.8, 13.7)	16.4 (15.8, 17.3)	34.6 (32.5, 35.1)	5.99 (4.50, 6.32)
						66.7%				
		Past HBV-infected group	26 * (21, 35)	23 * (19, 27)	8.0 * (6.0, 11.2)	37	13.2 (12.7, 13.7)	15.9 * (15.7, 16.4)	35.9 (34.0, 37.9)	5.13 * (4.15, 6.05)
						27.9%				
		Control group	28 * (20, 36)	22 * (19, 30)	7.2 * (5.2, 9.7)	27 *	12.9 (12.4, 13.3)	16.2 (15.7, 17.1)	34.6 (31.6, 36.4)	5.09 (4.43, 6.03)
						29.2%				

* *p* < 0.05 vs. the value before chemotherapy. Note: Data are presented as median (interquartile range) for continuous variables and n (%) for categorical variables, unless otherwise specified.

**Table 4:** Results of the linear mixed-effects models and generalized estimating equations examining factors associated with laboratory parameters during chemotherapy.

	Ln_ALT		Ln_AST		Ln_TBil		WBC		Liver Injury		PT		APTT		TT	
	F-Value	*p*-Value	F-Value	*p*-Value	F-Value	*p*-Value	F-Value	*p*-Value	χ^2^	*p*-Value	F-Value	*p*-Value	F-Value	*p*-Value	F-Value	*p*-Value
Fixed Effects																
Intercept	10,037.60	<0.001 *	14,868.90	<0.001 *	3752.90	<0.001 *	1380.80	<0.001 *	31.18	<0.001 *	34,775.23	<0.001 *	27,352.26	<0.001 *	16,306.96	<0.001 *
Age	48.66	<0.001 *	12.04	0.001 *	5.75	0.017 *	1.78	0.18	109.71	<0.001 *	38.23	<0.001 *	35.34	<0.001 *	40.76	<0.001 *
HBV Infection Status	14.73	<0.001 *	27.40	<0.001 *	3.64	0.026 *	0.42	0.66	24.09	<0.001 *	11.08	<0.001 *	5.38	0.032 *	3.20	0.041 *
Chemotherapy Regimen	1.66	0.19	6.57	0.038 *	6.26	0.002 *	7.41	0.001 *	3.86	0.15	3.41	0.033 *	0.87	0.43	1.05	0.35
Chemotherapy Cycle	309.12	<0.001 *	177.51	<0.001 *	117.91	<0.001 *	11.26	<0.001 *	351.38	<0.001 *	0.81	0.58	3.75	<0.131	6.48	<0.001 *
Chemotherapy Cycle × HBV Status	7.35	<0.001 *	7.20	<0.001 *	1.10	0.35	0.86	0.61	96.19	<0.001 *	0.52	0.92	2.02	0.035 *	1.96	0.017 *
Chemotherapy Cycle × Regimen	14.41	<0.001 *	15.32	<0.001 *	38.93	<0.001 *	3.08	<0.001 *	40.85	<0.001 *	2.77	<0.001 *	1.13	0.28	0.93	0.52
Chemotherapy Cycle × Regimen × HBV Status	1.68	0.12	2.30	<0.001 *	0.72	0.87	1.50	0.039 *	15.31	<0.039 *	0.64	0.78	1.21	0.35	0.73	0.68
Random Effects [Estimate]																
Intercept (Subject Variance)	0.13	<0.001 *	-	-	-	-	-	-	-	-	-	-	-	-	-	-
Residual (Variance)	0.16	<0.001 *	-	-	-	-	-	-	-	-	-	-	-	-	-	-

* *p* < 0.05.

**Table 5:** Dynamic changes in liver function parameters and risk of liver injury during chemotherapy in HBV-infected patients.

Cycles of Chemotherapy	ALT		AST		TBIL		Incidence of Liver Injury	
	HBV-Infected Group	*p*-Value	HBV-Infected Group	*p*-Value	HBV-Infected Group	*p*-Value	HBV-Infected Group	*p*-Value
1	0%	-	0%	-	0%	-	1	-
2	−9.7% * (−15.6%, −3.3%)	0.003	−4.5% (−9.2%, +0.4%)	0.163	−1.4% (−7.1%, +4.7%)	0.672	0.70 (0.49, 1.01)	0.054
3	+5.5% (−1.5%, +13.1%)	0.125	+9.6% * (+4.2%, +15.4%)	0.012	+0.2% (−5.5%, +6.5%)	0.731	1.13 (0.77, 1.65)	0.535
4	+16.7% * (+8.9%, +25.0%)	0.000	+14.4% * (+8.6%, +20.3%)	0.001	−2.6% (−8.3%, +3.6%)	0.285	1.56 * (1.06, 2.29)	0.025
5	+3.4% (−0.6%, +12.0%)	0.225	+5.8% * (+2.4%, +11.4%)	0.045	−4.1% (−9.8%, +2.0%)	0.128	1.28 *(1.09, 1.75)	0.031
6	+3.0% (−3.9%, +10.6%)	0.399	+6.6% * (+1.2%, +12.4%)	0.021	−7.3% * (−13.0%, −1.4%)	0.028	1.08 (0.74, 1.57)	0.696
7	−10.9% * (−18.6%, −2.5%)	0.012	−3.6% (−9.9%, +3.0%)	0.103	−4.1% (−11.5%, +3.9%)	0.117	0.77 (0.47, 1.26)	0.294
8	−15.2% * (−22.6%, −7.2%)	0.000	−8.7% * (−14.6%, −2.4%)	0.017	−6.9% (−14.0%, +0.8%)	0.069	0.66 (0.40, 1.09)	0.106

* *p* < 0.05.

**Table 6:** Dynamic changes in liver function parameters and risk of liver injury during chemotherapy in past HBV-infected patients.

Cycles of Chemotherapy	ALT		AST		TBIL		Incidence of Liver Injury	
	Past HBV-Infected Group	*p*-Value	Past HBV-Infected Group	*p*-Value	Past HBV-Infected Group	*p*-Value	Past HBV-Infected Group	*p*-Value
1	0%	-	0%	-	0%	-	1	-
2	−2.6% (−6.1%, +1.2%)	0.177	−1.9% (−4.6%, +0.9%)	0.415	−0.7% (−3.9%, +2.6%)	0.338	0.92 (0.74, 1.14)	0.457
3	−4.7% * (−8.2%, −1.0%)	0.013	−1.5% (−4.1%, +1.3%)	0.572	−0.2% (−3.0%, +3.6%)	0.761	0.84 (0.67, 1.05)	0.124
4	−1.9% (−5.5%, +2.0%)	0.326	−1.2% (−3.9%, +1.6%)	0.637	−2.7% (−5.8%, +0.6%)	0.085	0.90 (0.72, 1.12)	0.337
5	−2.3% (−6.0%, +1.5%)	0.235	+0.2% (−2.6%, +3.1%)	0.527	−0.8% (−4.1%, +2.6%)	0.351	0.91 (0.73, 1.14)	0.438
6	−1.3% (−5.0%, +2.6%)	0.525	+0.3% (−2.6%, +3.3%)	0.327	−1.2% (−4.5%, +2.2%)	0.176	0.90 (0.72, 1.12)	0.367
7	−2.6% (−7.2%, +2.3%)	0.299	−0.3% (−3.8%, +3.4%)	0.658	+1.3% (−3.0%, +5.8%)	0.106	0.88 (0.68, 1.14)	0.323
8	−4.2% (−8.9%, +0.6%)	0.087	−3.0% (−6.4%, +0.7%)	0.136	−0.3% (−4.6%, +4.1%)	0.257	0.83 (0.64, 1.08)	0.169

* *p* < 0.05.

## Data Availability

All raw data were stored in the computer medical record system of Chongqing Breast Cancer Center and the Health Management Center of the First Affiliated Hospital of Chongqing Medical University. To protect the privacy of the research participants, the original data will not be made public. However, you can contact the corresponding author upon reasonable request to obtain the data for this study.

## Supplementary Materials

The following supporting information can be downloaded at: https://www.mdpi.com/article/10.3390/cancers17213574/s1, Table S1: Comprehensive longitudinal data of laboratory parameters and liver injury incidence across all chemotherapy cycles, stratified by HBV serological status and regimen.

## Abbreviations

The following abbreviations are used in this manuscript:

HBV	hepatitis B virus
HBVr	hepatitis B virus reactivation
ALT	alanine Aminotransferase
AST	aspartate Aminotransferase
TBil	total bilirubin
PT	prothrombin time
TT	thrombin time
APTT	activated partial thromboplastin time
LMMs	Linear mixed-effects models
GEE	Generalized estimating equations
HBsAg	hepatitis B surface antigen
HBcAb	hepatitis B core antibody
HBeAb	hepatitis B e antibody
HBeAg	hepatitis B e antigen
HBsAb	hepatitis B surface antibody
ELISA	enzyme-linked immunosorbent assay
TRFIA	time-resolved fluoroimmunoassay
cccDNA	covalently closed circularDNA
