# Supplementary material for: The Impact of Different Hepatitis B Virus Serological Statuses on the Safety of Different Chemotherapy Regimens in Female Breast Cancer Patients: A Within-Subject Longitudinal Study
Source: Cancers (Basel). 2025 Nov 5;17(21):3574. doi: 10.3390/cancers17213574 (PMC12607424; doi:10.3390/cancers17213574)
Supplement: Supplementary file 1 [file cancers-17-03574-s001.zip › cancers-3917536-supplementary.pdf]

Table S1. Comprehensive Longitudinal Data of Laboratory Parameters and Liver Injury Incidence Across All Chemotherapy Cycles, Stratified by HBV Serological Status and Regimen.

Chemotherapy Regimen	Cycles of Chemotherapy	Serological Status of HBV	ALT (U/L)	AST (U/L)	TBiL（μmol/L）	Abnormal Liver Function	PT（s）	TT（s）	APTT（s）	WBC（10^9/L）
TEC（2731,59.8%）	1	HBV-infected group	19（13,28）	20（16,25）	10.5（8.0,13.4）	44 18.7%	13.0（12.6,13.6）	16.6（16.1,17.1）	35.7(33.1,37.8)	5.51（4.48,6.47）
		Past HBV-infected group	17（12,25）	18（15,22）	9.9（7.3,12.9）	221 14.1%	12.8（12.4,13.3）	16.6（16.0,17.2）	35.5(33.2,37.8)	5.57（4.67,6.55）
		Control group	16（11,24）	18（15,22）	9.7（7.0,13.0）	133 13.0%	12.8（12.4,13.3）	16.5（16.0,17.1）	35.8(33.5,38.1)	5.56（4.66,6.60）
	2	HBV-infected group	30*（22,39）	23*（18,28）	6.8*（5.0,9.2）	87* 37.0%	13.1（12.7,13.5）	16.1*（15.6,16.6）	33.6*(32.4,36.5)	5.27（4.06,6.57）
		Past HBV-infected group	28*（21,38）	21 *（17,27）	6.7*（4.9,9.0）	489* 32.4%	12.9（12.4,13.4）	16.1*（15.6,16.7）	34.6*(32.4,37.3)	5.78*（4.46,7.28）
		Control group	28*（21,39）	21*（18,27）	6.8*（4.8,9.0）	314* 31.7%	12.9（12.4,13.3）	15.9*（15.6,16.4）	35.4(33.4,37.8)	5.65*（4.58,7.15）
	3	HBV-infected group	33*（26,49）	26*（21,36）	7.6*（5.2,9.8）	108* 47.6%	13.3（12.7,13.5）	15.8*（15.3,16.5）	35.2(32.4,37.1)	5.10*（4.11,6.27）
		Past HBV-infected group	28*（21,38）	22*（18,28）	7.3*（5.3,9.5）	484* 32.4%	13.0*（12.6,13.3）	16.1*（15.5,16.6）	35.2(32.9,37.6)	5.28*（4.27,6.56）
		Control group	28*（22,40）	22*（18,29）	7.1*（5.3,9.5）	336* 33.7%	13.0*（12.6,13.4）	16.0*（15.5,16.6）	35.7(33.6,38.4)	5.30*（4.31,6.63）
	4	HBV-infected group	40*（27,55）	29*（22,41）	7.4*（5.4,10.1）	138* 60.0%	13.0（12.5,13.7）	16.1*（15.5,16.7）	36.1(34.4,38.9)	4.92*（3.79,6.22）
		Past HBV-infected group	29*（22,41）	23*（19,31）	7.2*（5.3,9.3）	558* 37.4%	13.0（12.5,13.4）	16.1*（15.6,16.5）	35.3(32.8,37.5)	5.07*（4.09,6.43）
		Control group	30*（23,43）	24*（19,31）	7.4*（5.4,9.7）	391* 39.7%	12.9（12.4,13.4）	16.1*（15.5,16.5）	36.2(33.9,38.0)	5.00*（4.03,6.32）
	5	HBV-infected group	33*（25,48）	26 *（21,33）	6.9*（4.9,9.5）	103* 45.5%	12.9（12.5,13.5）	15.8*（15.4,16.5）	35.2(32.5,37.5)	4.16*（3.12,5.42）
		Past HBV-infected group	28*（20,39）	23*（18,30）	6.7*（4.9,9.0）	514* 35.8%	12.7*（12.2,13.1）	16.0*（15.5,16.5）	35.0*(33.0,37.0)	4.42*（3.36,5.77）
		Control group	29*（21,41）	23*（18,31）	6.7*（4.9,9.0）	375* 38.9%	12.7*（12.2,13.1）	15.9*（15.4,16.5）	35.6(33.2,37.6)	4.37*（3.34,5.85）
	6	HBV-infected group	32*（25,46）	27 *（22,34）	6.8*（5.0,9.0）	100* 44.8%	13.2（12.8,13.7）	16.2（15.7,17.2）	35.4(33.0,38.7)	4.75*（3.64,5.76）
		Past HBV-infected group	30*（22,42）	25*（20,32）	6.9*（5.1,9.0）	569* 39.2%	13.0*（12.5,13.4）	16.1*（15.6,16.6）	35.1(32.9,37.2)	4.71*（3.70,5.95）
						402*				

		Control group	30*（23,44）	24*（19,32）	6.7*（4.9,9.1）	41.2%	13.0（12.5,13.3）	16.0*（15.6,16.7）	35.8(33.7,37.9)	4.62*（3.70,5.94）
		HBV-infected group	18（14,25）	20（16,22）	9.7（7.6,13.2）	15 16.0%	13.0（12.5,13.4）	16.6（16.1,17.2）	35.8(33.8,37.2)	5.44（4.62,6.20）
	1	Past HBV-infected group	15（11,23）	17（14,21）	9.2（6.9,12.3）	67 10.5%	12.9（12.4,13.3）	16.5（16.0,17.1）	35.6(33.3,37.8)	5.52（4.65,6.49）
		Control group	15（11,23）	17（14,21）	9.0（6.8,11.7）	54 12.4%	12.8（12.4,13.2）	16.5（15.9,17.1）	35.7(33.3,37.7)	5.38（4.54,6.30）
		HBV-infected group	27*（21,34）	22*（19,26）	6.2*（4.2,9.3）	23 25.0%	13.2（12.8,13.7）	16.2（15.8,17.3）	34.0(32.5,35.1)	5.30（4.05,6.41）
	2	Past HBV-infected group	25*（19,35）	21*（17,26）	5.7*（4.0,7.9）	165* 27.4%	13.2*（12.7,13.6）	16.2*（15.7,16.8）	35.1(32.7,37.6)	5.21*（4.12,6.52）
		Control group	26*（19,38）	21*（17,28）	5.7*（4.0,7.8）	128* 30.5%	13.2*（12.6,13.6）	16.0*（15.6,16.5）	35.9(33.1,39.0)	5.31（4.22,6.64）
		HBV-infected group	31*（23,44）	25*（21,33）	7.0*（4.6,9.0）	34* 38.8%	13.1（12.9,13.8）	16.6*（16.1,17.1）	34.6(32.9,36.9)	4.84（4.02,6.09）
	3	Past HBV-infected group	26*（20,36）	22*（18,29）	6.2*（4.2,8.3）	170* 28.3%	13.2*（12.7,13.5）	16.1*（15.7,16.7）	35.6(33.2,38.5)	4.86*（3.89,6.15）
		Control group	26*（20,39）	22*（18,29）	5.7*（4.2,8.1）	131* 32.5%	13.2*（12.7,13.5）	15.8*（15.5,16.4）	36.3(33.4,38.5)	4.83*（3.68,6.03）
EC-T(H)（1139,25.0%）		HBV-infected group	35*（27,47）	28*（22,38）	7.2*（4.6,9.0）	46* 53.1%	13.3（13.0,13.9）	16.0*（15.7,16.6）	34.2(33.3,36.0)	4.91*（4.05,6.22）
	4	Past HBV-infected group	30*（22,42）	25*（20,32）	6.5*（4.6,8.6）	238* 40.5%	13.0（12.6,13.5）	16.1*（15.7,16.7）	35.1(32.8,37.5)	4.67*（3.77,5.75）
		Control group	28*（20,44）	24*（19,33）	6.2*（4.5,8.7）	145* 36.2%	13.1（12.6,13.6）	15.9*（15.4,16.4）	35.2(33.3,37.9)	4.47*（3.76,5.61）
		HBV-infected group	37*（27,52）	30*（24,40）	6.4*（4.9,8.5）	53* 62.2%	13.2（12.5,13.6）	16.4（16.0,16.8）	33.9(33.3,36.5)	4.47*（3.52,5.77）
	5	Past HBV-infected group	32*（23,47）	26*（21,36）	6.6*（4.7,8.6）	269* 46.3%	12.9（12.5,13.3）	16.2*（15.7,16.6）	35.0(32.8,36.9)	4.36*（3.44,5.52）
		Control group	30*（21,46）	25*（20,33）	6.4*（4.6,8.6）	161* 40.2%	12.9（12.5,13.3）	16.0*（15.4,16.6）	35.3(32.9,37.9)	3.93*（3.20,5.04）
		HBV-infected group	41*（30,63）	33*（26,42）	7.5*（5.7,9.9）	59* 71.4%	13.3（13.0,13.5）	16.0（15.6,16.6）	34.5(33.1,36.0)	4.57*（3.68,5.74）
	6	Past HBV-infected group	34*（26,48）	27*（21,35）	7.6*（5.7,10.0）	297* 51.0%	13.0（12.5,13.4）	16.2*（15.7,16.7）	35.1(33.2,37.3)	4.94*（4.12,6.10）
		Control group	34*（25,49）	27*（22,34）	7.8*（5.9,10.1）	208* 50.4%	12.8（12.5,13.5）	16.0*（15.6,16.7）	35.6(33.8,38.4)	4.78*（4.02,6.03）
	7					46*				

		HBV-infected group	35*（27,46）	29*（24,36）	7.2*（6.2,9.0）	54.7%	12.8（12.5,13.5）	16.2（15.7,16.7）	34.8(33.6,36.0)	4.44*（3.50,5.62）
		Past HBV-infected group	33*（25,45）	26*（21,33）	7.4*（5.6,9.9）	271* 47.9%	13.0（12.5,13.3）	16.4*（15.9,16.8）	35.5(33.9,38.6)	4.97*（4.02,5.96）
		Control group	33*（24,45）	26*（21,33）	7.4*（5.5,9.7）	191* 48.3%	12.9（12.4,13.4）	16.2*（15.7,16.6）	35.6(33.4,38.4)	4.71*（3.92,5.91）
	8	HBV-infected group	33*（26,44）	27*（23,33）	7.3*（5.6,9.7）	39* 45.5%	13.3（12.6,13.5）	16.5（16.2,17.0）	35.0(32.2,36.9)	4.40*（3.92,5.60）
		Past HBV-infected group	32*（24,43）	26*（21,32）	7.3*（5.4,9.9）	243* 43.9%	12.9（12.4,13.3）	16.4*（15.9,17.0）	35.4(33.2,37.7)	4.94*（4.08,5.92）
		Control group	32*（24,44）	26*（21,32）	7.7*（5.8,9.6）	171* 44.4%	12.8（12.5,13.3）	16.2*（15.8,16.8）	35.4(33.5,38.0)	4.75*（4.00,5.79）
TC（217，4.8%）	1	HBV-infected group	24（13,31）	20（15,23）	9.6（7.2,15.6）	1 11.1%	13.0（12.5,13.4）	16.6（16.1,17.2）	35.8(33.8,37.2)	6.33（5.08,6.90）
		Past HBV-infected group	16（12,23）	19（16,23）	9.3（6.9,13.1）	26 18.6%	12.8（12.3,13.1）	16.7（16.0,17.2）	35.1(33.7,37.6)	5.58（4.66,6.42）
		Control group	16（12,21）	17（15,22）	8.8（6.7,11.3）	11 11.7%	12.9（12.4,13.4）	16.4（16.0,17.1）	35.8(33.7,38.1)	5.57（4.74,6.74）
	2	HBV-infected group	30（24,39）	25（19,29）	10.2（6.7,11.9）	2 22.2%	13.2（12.6,13.6）	16.3（15.8,17.3）	34.8(33.5,35.1)	5.43（3.85,6.51）
		Past HBV-infected group	25*（20,38）	21（17,27）	7.7（5.8,11.7）	42* 31.0%	13.2*（12.8,13.6）	16.5（16.1,16.8）	35.9(33.9,37.6)	5.18（4.19,6.67）
		Control group	20*（25,35）	21*（18,26）	7.8*（6.0,10.6）	26* 28.7%	13.1（12.4,13.6）	16.1（15.5,16.8）	33.6(31.6,36.7)	5.79（4.76,6.70）
	3	HBV-infected group	44（28,53）	29*（27,36）	8.2（6.4,14.7）	6* 66.7%	13.1（12.7,13.5）	16.2*（15.7,17.4）	34.9(33.6,35.1)	5.48（3.91,6.74）
		Past HBV-infected group	26*（21,36）	22*（18,27）	8.4（5.8,10.8）	38 28.6%	13.1（12.6,13.7）	16.2*（15.7,16.6）	35.4(34.5,37.1)	5.06（4.21,6.12）
		Control group	26*（21,38）	22*（19,28）	8.0（5.8,10.0）	26* 28.7%	13.1（12.8,13.7）	16.0（15.4,16.5）	35.1(33.6,36.3)	5.44（4.63,6.79）
	4	HBV-infected group	56*（44,57）	39*（28,53）	11.1（9.4,14.2）	6* 66.7%	13.0（12.8,13.7）	16.4（15.8,17.3）	34.6(32.5,35.1)	5.99（4.50,6.32）
		Past HBV-infected group	26*（21,35）	23*（19,27）	8.0*（6.0,11.2）	37 27.9%	13.2（12.7,13.7）	15.9*（15.7,16.4）	35.9(34.0,37.9)	5.13*（4.15,6.05）
		Control group	28*（20,36）	22*（19,30）	7.2*（5.2,9.7）	27* 29.2%	12.9（12.4,13.3）	16.2（15.7,17.1）	34.6(31.6,36.4)	5.09（4.43,6.03）

* $p < 0.05$ vs the value before chemotherapy.
